# Numeracy and Literacy Independently Predict Patients’ Ability to Identify Out-of-Range Test Results

**DOI:** 10.2196/jmir.3241

**Published:** 2014-08-08

**Authors:** Brian J Zikmund-Fisher, Nicole L Exe, Holly O Witteman

**Affiliations:** ^1^Department of Health Behavior and Health EducationUniversity of MichiganAnn Arbor, MIUnited States; ^2^Department of Internal MedicineUniversity of MichiganAnn Arbor, MIUnited States; ^3^Center for Bioethics and Social Sciences in MedicineUniversity of MichiganAnn Arbor, MIUnited States; ^4^Risk Science CenterUniversity of MichiganAnn Arbor, MIUnited States; ^5^Department of Family and Emergency MedicineFaculty of MedicineUniversité LavalQuébec City, QCCanada; ^6^Office of Education and Continuing Professional DevelopmentFaculty of MedicineUniversité LavalQuébec City, QCCanada; ^7^Research Centre of the CHU de QuébecQuébec City, QCCanada

**Keywords:** numeracy, literacy, patient education as topic, electronic health records

## Abstract

**Background:**

Increasing numbers of patients have direct access to laboratory test results outside of clinical consultations. This offers increased opportunities for both self-management of chronic conditions and advance preparation for clinic visits if patients are able to identify test results that are outside the reference ranges.

**Objective:**

Our objective was to assess whether adults can identify laboratory blood test values outside reference ranges when presented in a format similar to some current patient portals implemented within electronic health record (EHR) systems.

**Methods:**

In an Internet-administered survey, adults aged 40-70 years, approximately half with diabetes, were asked to imagine that they had type 2 diabetes. They were shown laboratory test results displayed in a standard tabular format. We randomized hemoglobin A_1c_ values to be slightly (7.1%) or moderately (8.4%) outside the reference range and randomized other test results to be within or outside their reference ranges (ie, multiple deviations). We assessed (1) whether respondents identified the hemoglobin A_1c_ level as outside the reference range, (2) how respondents rated glycemic control, and (3) whether they would call their doctor. We also measured numeracy and health literacy.

**Results:**

Among the 1817 adult participants, viewing test results with multiple deviations increased the probability of identifying hemoglobin A_1c_ values as outside the reference range (participants with diabetes: OR 1.47, 95% CI 1.12-1.92, *P*=.005; participants without diabetes: OR 1.50, 95% CI 1.13-2.00, *P*=.005). Both numeracy and health literacy were significant predictors of correctly identifying out-of-range values. For participants with diabetes, numeracy OR 1.32 per unit on a 1-6 scale (95% CI 1.15-1.51, *P*<.001) and literacy OR 1.59 per unit of a 1-5 scale (95% CI 1.35-1.87, *P*<.001); for participants without diabetes, numeracy OR 1.36 per unit (95% CI 1.17-1.58, *P*<.001) and literacy OR 1.33 per unit (95% CI 1.12-1.58, *P*=.001). Predicted probabilities suggested 77% of higher numeracy and health literacy participants, but only 38% of lower numeracy and literacy participants, could correctly identify the hemoglobin A_1c_ levels as outside the reference range. Correct identification reduced perceived blood glucose control (mean difference 1.68-1.71 points on a 0-10 scale, *P*<.001). For participants with diabetes, increased health literacy reduced the likelihood of calling one’s doctor when hemoglobin A_1c_=7.1% (OR 0.66 per unit, 95% CI 0.52-0.82, *P*<.001) and increased numeracy increased intention to call when hemoglobin A_1c_=8.4% (OR 1.36 per unit, 95% CI 1.10-1.69, *P*=.005).

**Conclusions:**

Limited health literacy and numeracy skills are significant barriers to basic use of laboratory test result data as currently presented in some EHR portals. Regarding contacting their doctor, less numerate and literate participants with diabetes appear insensitive to the hemoglobin A_1c_ level shown, whereas highly numerate and literate participants with diabetes appear very sensitive. Alternate approaches appear necessary to make test results more meaningful.

## Introduction

Increasing numbers of patients have direct access to laboratory test results outside of clinical consultations via patient portals implemented within electronic health record (EHR) systems. Patient access to such records was included in the Centers for Medicare and Medicaid Services (CMS) Meaningful Use of Health Information Technology criteria from 2010 [1], and already exists in some large health care systems. Strong federal incentives supporting adoption of electronic medical record systems will likely significantly increase the availability of patient portals in the future.

Patients use such systems to view medical test results and value being able to do so [2-4]. Direct patient use of test data is consistent with trends toward patient-centered approaches to care, patient engagement, and the medical home concept, all of which encourage greater patient involvement in both medical decision making and health self-management [5-7]. In that manner, patient access to such health data promotes a transfer of some of the responsibility for health management from care providers to the patients themselves [8]. Patient access is also congruent with the trend for people to actively gather, manage, and analyze their personal data (eg, the “quantified self” movement) [9]. Perhaps most importantly, there is an ethical imperative to provide easy access to patients who want it [10].

Patients want to be notified of laboratory test results, regardless of whether the findings were normal or abnormal [11], because failures to inform patients of test results are unfortunately all too common, even for abnormal or otherwise actionable test results [12]. Direct access enables patients to seek out their results by themselves, thereby providing a second opportunity for identifying actionable results and preventing unnecessary harm.

Test result data can also enable patients to better prepare for clinic visits by focusing their attention on test results that are abnormal or of concern. This knowledge could lead patients to prepare questions or seek out relevant information before the visit. Such preparation benefits patients, but it also benefits the health care system by making visits more efficient [1].

Patients can also use test results to improve self-management of their current health conditions [1,13]. For example, a person with diabetes could both assess her current status and identify long-term trends in her blood glucose control. She could use such data to determine whether her current health management efforts (eg, behavior programs, medications) are working. Such information offers the potential to increase patient activation and the likelihood of engaging in particular treatment or health behaviors [1].

Achievement of these potential benefits, however, requires patients to perform a simple, yet critical, task: to be able to correctly identify which test results are out of range (ie, outside the reference ranges) from the (usually) much larger set of data provided. Unfortunately, there are several reasons to suspect that many patients will have difficulty with this task when the test results are displayed in the tabular format currently used in many interface designs.

First, many patients have limited health literacy, which inhibits their ability to interpret the health information they read and use that information to manage their health [14-16]. For example, low health literacy is associated with less knowledge about the medications one is taking [17], being less able to read and understand medication labels [18], unintentional nonadherence to hospital discharge instructions [19], and increased mortality [20,21]. Health literacy affects patient use of laboratory test results in 2 ways. First, lack of attention to issues of health literacy when designing patient portals limits the accessibility of such tools and, therefore, limits their impact among those who might most benefit from them. Even restricting analysis to people with Internet access, lower health literacy is associated with a lower likelihood of logging into a patient portal in the first place [22]. Indeed, patient portal use is lower among the more vulnerable populations [23]. Second, health literacy affects patients’ abilities to gather background and contextual information (eg, about what a test is, what values are normal or concerning) necessary to cognitively evaluate the meaning of a test result in relation to their health.

Second, many patients also have lower numeracy skills (ie, poor ability to use and draw meaning from numbers) [24,25]. Although some measures of health literacy (eg, Test of Functional Health Literacy in Adults, TOFHLA) include assessments of what is variably termed numerical ability or quantitative literacy, there is growing evidence that numeracy is a distinct construct that is particularly relevant to data interpretation tasks. Numeracy predicts people’s ability to read nutrition labels, calculate medication dosages, maintain anticoagulation control, and maintain glycemic control better than measures of health literacy do [26-29]. Patients with lower numeracy skills may lack the capacity to interpret test outcome data in some current presentations. In addition, numeracy appears necessary for people to develop emotional responses to data [30]. This is problematic given the large amount of theoretical and experimental evidence that emotions are both integral to risk perceptions and necessary for effective decision making [31-34]. As a result, less numerate patients are unlikely to know how to use medical test results if they cannot get a feeling of “goodness” or “badness” from the data [30].

Without careful design that attends to issues of health literacy and numeracy, presentations of laboratory test results (whether in patient portals or via a clinician’s office) could be of little use to less literate and numerate patients. Although some initiatives have used cues such as color to help patients identify out-of-range values [35], often laboratory results are shown in the same tabular format that is provided to clinicians. These tables present a dozen or more tests simultaneously, usually labeled with unfamiliar abbreviations, reported in unfamiliar units, and lacking guidance as to whether higher numbers represent more positive or negative outcomes. Unfortunately, less numerate people have particular difficulty identifying decision-relevant information out of larger sets of data [36]. Therefore, the sheer volume of information available through patient portals is particularly challenging for the less numerate [8].

We designed an experimental study to assess the degree that adults, especially those with lower numeracy and/or lower health literacy, are able or not able to identify out-of-range values in prototypical medical test result displays. Participants viewed multiple panels of test results typical of what would be ordered for ongoing management of a person with type 2 diabetes and were asked to (1) identify all values outside the reference range, (2) assess the degree of blood glucose control represented by those results, and (3) identify whether they would call their doctor regarding these results. To test patient sensitivity to variations in test results, we experimentally varied 2 factors: (1) hemoglobin A_1c_ levels were mildly or moderately elevated and (2) other test results were within or outside their reference ranges. To enable assessment of the role of numeracy and health literacy skills on people’s ability to complete these tasks accurately, all participants completed validated measures of both constructs.

## Methods

### Participants

We recruited a stratified random sample of US adults aged 40-70 years from a panel of Internet users administered by Survey Sampling International (SSI, Shelton, CT, USA), which recruits panel members through various opt-in methods. To ensure demographic diversity (although not representativeness) and offset variations in response rates, we drew subsamples by both age and race (thereby approximating the distributions of these characteristics in the US population). We also drew separate subsamples by experience with diabetes: We specified that approximately half of completed surveys be from panel participants who had previously indicated that they had diabetes (and hence might have had greater knowledge about hemoglobin A_1c_ tests) and half from people without personal experience with diabetes (who might be more similar to newly diagnosed patients). The number of email invitations in each subsample was dynamically adjusted until quotas were achieved.

Selected panel members received email invitations with a personalized link (tracked to prevent duplicates) and nonresponders received 1 reminder email. Those who clicked on the link then viewed an introductory page that provided information about the estimated length of the survey (10 to 15 minutes), the purpose of the study, and affiliation and contact information for the investigators before taking the participant to the main study materials. We recruited for a 2-week period in January 2013. On completion, participants were entered into instant-win contests and regular draws administered by SSI for modest prizes.

### Design

**Figure 1:**
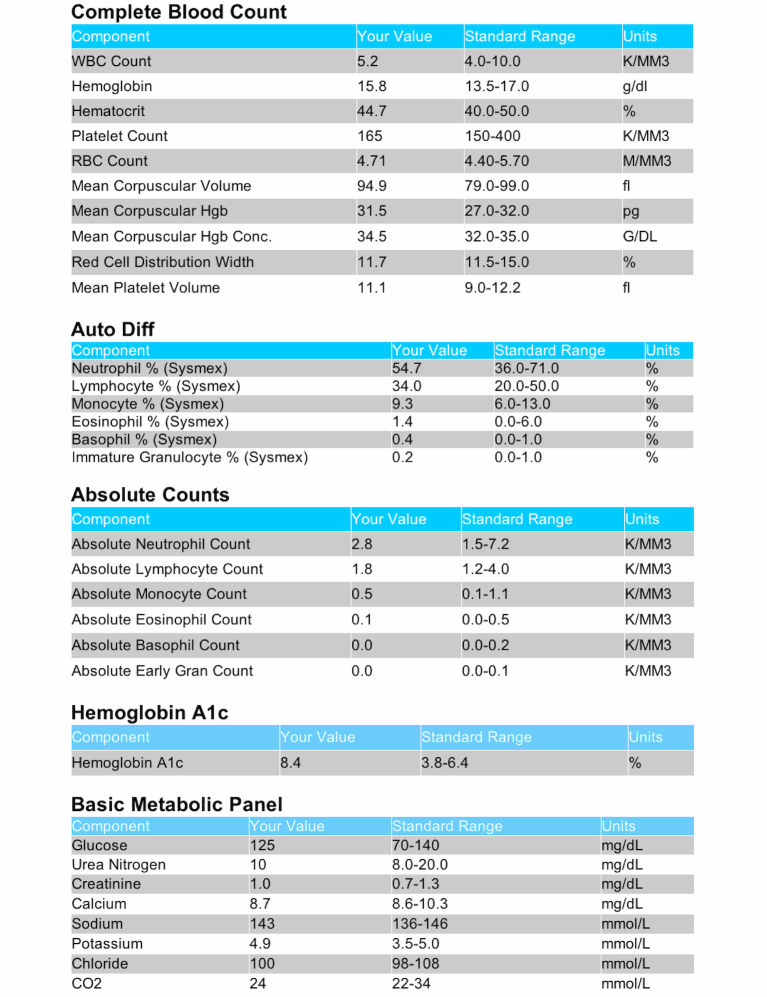
Screenshot of the test results display (hemoglobin A1c=8.4%, single deviation condition).

Participants were asked to imagine that they were diagnosed with type 2 diabetes, had been maintaining good blood glucose control with a previous hemoglobin A_1c_ test result of 6.8%, and had an explicit goal of maintaining hemoglobin A_1c_ values below 7%. Participants were then asked to imagine that they were viewing the results of a set of blood tests (complete blood cell count, CBC; hemoglobin A_1c_; and renal panel) that had been ordered between doctor’s visits. Following the format currently implemented in the patient portal of a major academic medical center, all tables showed test values, standard ranges, and units, but did not show indicators for high or low values (the medical center includes high/low indicators in clinician interfaces but omits them from the patient interface). As shown in Figure 1, all tests were presented on a single page grouped by panel per standard practice.

We manipulated the test results shown in a 2×2 factorial design. All participants viewed results that showed that hemoglobin A_1c_ was elevated above the standard range (reported as 3.8%-6.4%). We randomly varied the degree of A_1c_ elevation by randomizing participants to view a hemoglobin A_1c_ result of either 7.1% or 8.4%. Thus, both values should be identified as out of range, but only the 8.4% value is sufficiently high (and a large enough change from the previous value) to potentially warrant more timely attention. In addition, we independently varied whether all other reported results were within standard ranges (single deviation condition) or whether multiple results were out of range (multiple deviations condition). Participants in the multiple deviations condition saw tables with out-of-range values for white blood cell (WBC) count, platelet count, mean corpuscular hemoglobin (MCH), mean corpuscular hemoglobin concentration (MCHC), neutrophil %, lymphocyte %, monocyte %, absolute neutrophil count, and serum glucose. These values were either elevated or reduced to be consistent with a temporary viral infection.

### Outcome Measures

We asked participants to answer a series of questions about the test results display, which remained visible so that the questions would measure test understanding and interpretation, not recall.

Participants first rated their perceived blood glucose control based on the hypothetical scenario and data by answering the question “according to these test results, how well have you been keeping your blood sugars under control *over the last 3 months*?” (emphasis in original), with responses on an 11-point scale ranging from not at all well to extremely well.

The next question asked participants to “please mark which tests had results different than what they should be” (ie, had out-of-range values) using a set of checkboxes. If participants marked any tests as being out of range, they received a follow-up question asking them to “please rate how concerned you would be about each of these identified tests” on a 5-point scale from not at all concerned to extremely concerned. Our analysis focused on whether participants identified the hemoglobin A_1c_ value as out of range and their subsequent ratings of concern.

Next, we asked participants to indicate what they would do after reviewing the test results (with test results no longer visible). Participants chose from 3 options: (1) call or email doctor’s office and ask to speak with your doctor immediately, (2) call or email doctor’s office to see whether you can schedule an appointment with your doctor in the next few weeks (your next scheduled appointment is currently in 3 months), or (3) talk to your doctor about these results at your next appointment in 3 months.

We also asked 2 questions to measure perceived usefulness. Participants rated “how well did you understand what the test results said” on a 5-point scale from did not understand at all to understood completely and “how useful were these test results” on a 5-point scale from not at all useful to extremely useful.

### Individual Difference Measures

Participants next completed several individual difference measures that we hypothesized might interact with ability to interpret test result tables. Because ample evidence exists that even highly educated adults can have poor numeracy skills (ie, facility and comfort with quantitative health information such as risk statistics) [25,37,38], all study participants completed the Subjective Numeracy Scale (SNS) [39]. The SNS measures both perceived quantitative ability and preference for receiving information in numerical form and has previously been shown to correlate with the ability to recall and comprehend both textual and graphical risk communications [40,41]. A participant’s SNS score is calculated as his or her mean rating across the 8 SNS questions (after accounting for reverse coding) and ranges from 1 (least numerate) to 6 (most numerate). In addition, participants also completed Chew et al’s [42] 3-question measure of limited health literacy, which has been validated and shown to be highly correlated with other measures, such as the Rapid Estimate of Adult Literacy in Medicine (REALM) and Short Test of Functional Health Literacy in Adults (S-TOFHLA). Participants’ literacy score was the mean response for the 3 questions (after reverse coding 1 question) and ranges from 1 (least literate) to 5 (most literate).

Participants also completed standard demographic questions and indicated whether they were diagnosed previously with diabetes. This latter direct-response measure was used for analysis of the effect of diabetes experience instead of the information from SSI that had been used to guide the sampling process.

### Data Management

All data were collected anonymously using the Qualtrics online survey platform. Participants were identified and prevented from taking the survey multiple times via unique identification numbers provided by SSI within the redirected URL. The design, sampling process, data management procedures, and outcome measures received exempt status approval from the University of Michigan Health Sciences and Behavioral Sciences Institutional Review Board.

### Statistical Analyses

We performed separate analyses of data from participants who self-reported that they were diagnosed with diabetes and from nondiabetic participants. For each group, we conducted chi-square and logistic regression analyses of whether respondents identified the hemoglobin A1c value as out of range and whether they intended to call their doctor about the test results. We conducted *t* tests, correlation analyses, and linear regression analyses of both perceived blood glucose control and perceived usefulness of the test result displays. Regression analyses included indicator variables for experimental factors, education, and respondents’ health literacy and numeracy scale scores as continuous variables. Education was modeled as 2 indicator variables for (1) greater than high school education of some type, but no bachelor degree and (2) a bachelor or higher degree (each compared to a baseline group of high school education or lower). We also conducted additional analyses to determine whether the ability to correctly identify a test result as out of range mediated any effects of experimental or demographic predictors on perceptions of blood glucose control and intentions to call a doctor. All analyses were performed using Stata 12 (StataCorp LP, College Station, TX, USA), and all tests of significance were 2-sided and used alpha=.05.

## Results

### Participant Characteristics

In total, 1817 people aged between 40 and 70 years completed the survey. Participant characteristics are reported in Table 1. We observed a wide range of educational achievement with 31.36% (567/1808) of participants having a bachelor or higher college degree, but also 23.73% (429/1808) with an education level of high school or less.

Within our sample, the SNS numeracy measure showed high reliability (Cronbach alpha=.87), and the mean SNS score was 4.47 (SD 1.06, range 1.0-6.0). Mean score on the health literacy measure was 3.84 (SD 0.87, range 1-5), although the scale showed relatively weak reliability (Cronbach alpha=.54). These 2 measures were moderately correlated (*r*=.26), although 189 of 1799 (10.51%) participants indicated lower numeracy (SNS ≤4) and higher literacy (literacy ≥4) and 117 of 1799 (6.50%) participants had the reverse pattern of higher numeracy (SNS ≥5) and lower literacy (literacy ≤3).

**Table 1:** Participant characteristics (N=1817).

Characteristic and categories		n (%)^a^	Mean (SD)
**Age (years) (n=1814)**			54.2 (8.4)
	40-49	635 (35.01)	
	50-59	605 (33.35)	
	60-70	574 (31.64)	
**Sex (n=1814)**			
	Male	901 (49.67)	
	Female	913 (50.33)	
**Ethnicity (n=1807)**			
	Hispanic (any race)	170 (9.41)	
**Race** ^b^ **(n=1810)**			
	White	1407 (77.73)	
	African-American	280 (15.47)	
	All other	161 (8.90)	
**Education (n=1808)**			
	≤High school	429 (23.73)	
	Some college/trade	812 (44.91)	
	Bachelor/master/doctorate degree	567 (31.36)	
**Subjective Numeracy Scale Score (n=1804)**			4.47 (1.06)
	1.00-1.99	42 (2.33)	
	2.00-2.99	138 (7.65)	
	3.00-3.99	315 (17.46)	
	4.00-4.99	600 (33.26)	
	5.00-5.99	644 (35.70)	
	6.00	65 (3.60)	
**Limited Health Literacy Scale Score (n=1799)**			3.84 (0.87)
	1.00-1.99	19 (1.06)	
	2.00-2.99	219 (12.17)	
	3.00-3.99	649 (36.08)	
	4.00-4.99	576 (32.02)	
	5.00	336 (18.68)	
Participant with diabetes (n=1812)		971 (53.59)	

^a^ Reports results only for those respondents who completed each question or measure.

^b^ Respondents could indicate more than 1 race.

### Identification of Hemoglobin A1c Value as Out of Range

Overall, approximately half (931/1817, 51.24%) of participants correctly identified the hemoglobin A_1c_ value as being “different than what [it] should be.” Participants with diabetes were more likely to identify the out-of-range hemoglobin A_1c_ value than participants without diabetes were (participants with diabetes: 546/971, 56.2%; participants without diabetes: 384/841, 45.7%, χ^2^_1_=20.2, *P*<.001). Rates of correctly identifying out-of-range hemoglobin A_1c_ values were also significantly higher among participants in the multiple deviations condition versus those in the single deviation condition (multiple deviations: 499/898, 55.6%; single deviation: 432/919, 47.0%, χ^2^_1_=13.3, *P*<.001). The specific hemoglobin A_1c_ value reported had no effect on the likelihood of marking it as out of range (hemoglobin A_1c_=7.1%: 462/911, 50.7%; hemoglobin A_1c_=8.4%: 469/906, 51.8%; χ^2^_1_=0.2, *P*=.65).

Table 2 reports logistic regression analyses identifying predictors of correctly identifying hemoglobin A_1c_ values as out of range for participants with and without diabetes. The multivariate analysis confirms the significant effect of the multiple deviations condition for both groups of participants. However, the specific hemoglobin A_1c_ value shown was a significant predictor of correctly identifying it as out of range for participants with diabetes (more likely to mark if hemoglobin A_1c_=8.4% vs 7.1%). In addition, the regression analyses identified significant and independent effects of both participant numeracy and health literacy as well as weaker effects of education.

**Table 2:** Logistic regression results showing predictors of identifying hemoglobin A_1c_ levels as out of range for participants with and without diabetes.

Variable	Participants without diabetes (n=827)			Participants with diabetes (n=963)		
	OR	95% CI	*P*	OR	95% CI	*P*
Hemoglobin A_1c_ test result=8.4% (vs=7.1%)	0.85	0.64, 1.13	.26	1.38	1.05, 1.80	.02
Multiple deviations condition (vs single)	1.50	1.13, 2.00	.005	1.47	1.12, 1.92	.005
Education: high school or less	—	—	—	—	—	—
Education: >high school but <bachelor degree	1.28	0.88, 1.86	.20	1.17	0.83, 1.66	.36
Education: bachelor degree or higher	1.41	0.94, 2.11	.10	1.53	1.03, 2.27	.04
Subjective numeracy score (per unit, range 1-6)	1.36	1.17, 1.57	<.001	1.32	1.15, 1.51	<.001
Literacy score (per unit, range 1-5)	1.33	1.12, 1.58	.001	1.59	1.35, 1.87	<.001

**Figure 2:**
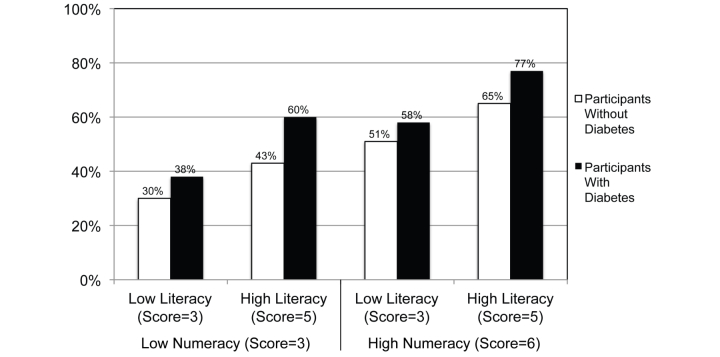
Predicted probabilities that participants with and without diabetes would correctly identify hemoglobin A1c test results as outside the standard range by lower versus higher literacy and numeracy levels.

To clarify the effect size of the experience with diabetes, numeracy, and health literacy effects, we calculated the predicted likelihood that participants with different combinations of lower versus higher numeracy and lower versus higher health literacy would mark hemoglobin A_1c_ levels as out of range, holding all other predictors to their mean values. We conducted this analysis separately for participants with and without diabetes. Although we recognize that numeracy and literacy are often at least moderately correlated in practice (in our sample: *r*=.26), the predicted probabilities help to clarify the independent and combined effects of these 2 factors over the range of possible patient skill levels. We defined lower numeracy or health literacy as a score of 3 on these scales (corresponding to the tenth to thirteenth percentile of the observed distribution for each measure) and higher numeracy as the scale maximums of 6 for numeracy and 5 for health literacy. As Figure 2 shows, the combined effect of lower health literacy and lower numeracy more than halves the probability of identifying out-of-range values (from 77% to 38% for participants with diabetes and from 65% to 30% for participants without diabetes).

### Perceptions of Blood Glucose Control

Perceptions of blood glucose control averaged in the middle of the 0-10 scale, but we observed substantial variance. When the displayed hemoglobin A_1c_ level was 7.1%, the mean perception of blood glucose control was 5.62 (SD 2.87) and did not vary significantly by diabetes diagnosis. However, when the displayed hemoglobin A1c level was 8.4%, not only were perceptions of control lower (mean 4.71, SD 3.17) but perceptions of the participants with diabetes were significantly lower than those of participants without diabetes (participants with diabetes: mean 4.40, SD 3.13; participants without diabetes: mean 5.05, SD 3.18; *t*_887_=3.07, *P*=.002).

Table 3 reports linear regressions predicting perceived blood glucose control among participants with and without diabetes. Because the act of identifying the hemoglobin A_1c_ test result as being outside the standard range is a likely precursor of perceiving one’s blood glucose as being less controlled, we included that variable as an independent predictor in addition to the previous model predictors.

Confirming our expectations, correctly identifying hemoglobin A_1c_ levels as out of range had a highly significant and large effect on participants’ ratings of their blood glucose control, reducing ratings of blood glucose control by 1.68-1.71 points on the 11-point scale (*P*<.001 for both). Also, viewing a hemoglobin A_1c_ test result of 8.4% instead of 7.1% also lowered perceptions of blood glucose control (*P*<.001 for both).

However, we also observed independent effects of health literacy and numeracy for participants without diabetes. Having higher health literacy decreased perceived glucose control by 0.54 points per unit on the 5-point literacy scale (*P*<.001), whereas higher numeracy increased perceived glucose control by 0.27 points per unit on the 6-point numeracy scale (*P*=.01).

The fact that health literacy and numeracy predicted correctly identifying the hemoglobin A_1c_ value as out of range raises the possibility that their effects on perceived blood glucose control might be partially mediated through that action. Yet, reduced models omitting the “marked A_1c_ as out-of-range” variable (not shown) were similar to those shown in Table 3. Among participants without diabetes, test result (hemoglobin A_1c_ value of 8.4% vs 7.1%) and health literacy remained highly significant, and numeracy was actually less significant (beta=0.15, *P*=.16). Among participants with diabetes, the effects of test result and high education remained highly significant, and the coefficient for health literacy became significant (beta=–0.35, *P*=.002). This suggests that correctly identifying hemoglobin A_1c_ levels as out of range may have partially mediated the effect of health literacy among participants with diabetes, but we saw no evidence of any mediation effects among participants without diabetes.

**Table 3:** Linear regression results showing predictors of perceived blood sugar control among participants with and without diabetes.

Variable	Participants without diabetes (n=820)		Participants with diabetes (n=953)	
	Coefficient	*P*	Coefficient	*P*
Hemoglobin A_1c_ test result=8.4% (vs 7.1%)	–0.65	.001	–1.08	<.001
Multiple alert condition (vs single alert)	–0.37	.07	–0.13	.46
Education: high school or less	—	—	—	—
Education: >high school but <bachelor degree	0.24	.37	–0.54	.03
Education: bachelor’s degree or higher	–0.35	.23	–0.76	.005
Subjective numeracy score (per unit, range 1-6)	0.27	.01	0.09	.33
Literacy score (per unit, range 1-5)	–0.54	<.001	–0.17	.11
Marked hemoglobin A_1c_ result as out of range	–1.68	<.001	–1.71	<.001
Constant	7.52		7.29	

### Behavioral Intentions Regarding Contacting a Doctor

Table 4 reports the proportion of respondents in each experimental condition who indicated that they would intend to call their doctor to discuss the laboratory test results, organized by participants with diabetes and those without. Most participants would call their doctor in all conditions (1218/1765, 69.01%), but in the single deviation condition, the intention to call was significantly lower when hemoglobin A_1c_ level was 7.1% vs 8.4% (A_1c_=7.1%: χ^2^_1_=15.4, *P*<.001; A_1c_=8.4%: χ^2^_1_=18.9, *P*<.001). However, in the multiple deviation condition, the difference in rates was significant (but only barely so) among participants with diabetes (χ^2^_1_=4.3, *P*=.04) and was nonsignificant among participants without diabetes (χ^2^_1_=0.1, *P*=.76).

**Table 4:** Proportion of respondents indicating they would call their doctor to discuss the laboratory test results (either immediately or rebook a set appointment to an earlier date/time) by diabetes diagnosis and experimental condition (N=1763).

Condition	Participants without diabetes (n=813)		Participants with diabetes (n=950)	
	A_1c_=7.1%	A_1c_=8.4%	A_1c_=7.1%	A_1c_=8.4%
Single deviation condition, n/n (%)	137/215 (63.7%)	178/221 (80.5%)	119/232 (51.3%)	160/225 (71.1%)
Multiple deviation condition, n/n (%)	138/186 (74.2%)	139/191 (72.8%)	167/253 (66.0%)	179/240 (74.6%)

We next report the results of logistic regression analyses of intent to call a doctor for participants with and without diabetes (Table 5). Within each table, we report separate analyses for the hemoglobin A_1c_=7.1% and 8.4% scenarios because they represent 2 distinct scenarios that should logically evoke different behaviors in participants and therefore might have fundamentally different predictors.

**Table 5:** Logistic regression results showing predictors of intent to call a doctor among study participants with and without diabetes.

Variable	Participants without diabetes						Participants with diabetes					
	A_1c_=7.1%			A_1c_=8.4%			A_1c_=7.1%			A_1c_=8.4%		
	OR	95% CI	*P*	OR	95% CI	*P*	OR	95% CI	*P*	OR	95% CI	*P*
Multiple deviations condition (vs single)	1.47	0.94, 2.29	.09	0.54	0.33, 0.88	.01	1.79	1.23, 2.62	.003	1.11	0.73, 1.70	.63
Education: high school or less	—	—	—	—	—	—	—	—	—	—	—	—
Education: >high school but <bachelor degree	0.81	0.46, 1.43	.47	1.45	0.78, 2.69	.24	1.08	0.66, 1.76	.77	0.85	0.49, 1.50	.58
Education: bachelor degree or higher	0.98	0.51, 1.87	.95	0.95	0.50, 1.81	.88	1.36	0.79, 2.36	.27	0.64	0.33, 1.25	.19
Subjective numeracy score (per unit, range 1-6)	1.09	0.87, 1.35	.46	1.17	0.92, 1.48	.20	0.94	0.77, 1.15	.53	1.36	1.10, 1.69	.005
Literacy score (per unit, range 1-5)	0.86	0.65, 1.12	.26	0.91	0.67, 1.23	.54	0.66	0.52, 0.82	<.001	0.92	0.71, 1.20	.55
Marked A_1c_ as out of range	1.98	1.26, 3.11	.003	3.28	1.91, 5.61	<.001	1.95	1.31, 2.89	.001	2.31	1.48, 3.61	<.001

Among participants without diabetes, correctly identifying hemoglobin A_1c_ levels as out of range was the primary predictor of whether the participant intended to call their doctor. However, we also saw an interesting pattern regarding the multiple deviations condition. Having other test results (beyond hemoglobin A_1c_) out of range tended (nonsignificantly) to increase the odds of calling the doctor if the hemoglobin A_1c_ level was 7.1% but significantly lowered the likelihood of calling the doctor if the hemoglobin A_1c_ level was 8.4%. It is unclear whether the latter effect reflects beliefs that the elevated hemoglobin A_1c_ level is not as concerning in the presence of the other nonnormal test results or simple confusion or distraction. In either case, neither health literacy nor subjective numeracy scores predicted intentions to call the doctor’s office among participants without diabetes. Omitting the “marked A_1c_ as out of range” variable had little effect on either regression. The only change of note was that, in the hemoglobin A_1c_ level equals 7.1% condition, the odds ratio for the multiple deviations condition increased slightly and became statistically significant (OR 1.59, 95% CI 1.03-2.45, *P*=.04).

Among study participants with diabetes, we saw a distinct pattern of results related to health literacy and numeracy even after controlling for the continued large and significant effect of having correctly identified hemoglobin A_1c_ levels as out of range (which was highly predicted by health literacy and numeracy). Among participants with diabetes presented with hemoglobin A_1c_=7.1% test results, increased health literacy significantly reduced the likelihood of calling one’s doctor. This is consistent with these individuals having absorbed the background knowledge provided in the scenario sufficiently to recognize that a 7.1% value is only mildly elevated in comparison to the previous value of 6.8% that was provided in the scenario and was very close to the 7% threshold that was stated explicitly as the patient’s goal level. However, numeracy skills were not associated with intentions to call the doctor.

Conversely, among participants with diabetes who were presented with hemoglobin A_1c_=8.4% test results (a value that is both significantly elevated on an absolute level and a much larger increase in reference to the previous test result cited in the scenario), health literacy had no effect on intentions to call their doctor. Instead, among participants with diabetes, it was increased numeracy skills that significantly increased their intentions to call the doctor’s office. This finding is consistent with the hypothesis that numeracy skills were associated with respondents’ ability to recognize not merely that the 8.4% value was out of range, but that the increase of less than 2 absolute percentage points nonetheless represented a substantial and concerning change worthy of action. Both this effect and the effect of health literacy when the A_1c_ test result was 7.1% remain essentially unchanged if “marking A1c as out of range” was removed from the regression equations.

**Figure 3:**
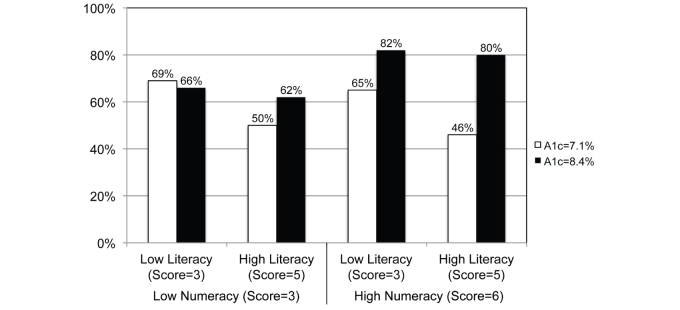
Predicted probabilities that participants with diabetes would call their doctor by reported hemoglobin A1c level and by lower versus higher literacy and numeracy levels.

The magnitude of both of these effects is illustrated via the predicted rates of intentions to call one’s doctor shown in Figure 3. What is clear from this figure is that health literacy and numeracy directly impacted sensitivity to the test results among participants with diabetes. Our analyses predicted that patients with diabetes with low numeracy skills and low health literacy would be just as likely to call their doctor when the hemoglobin A_1c_ levels were 7.1% or 8.4%. In contrast, our model predicted that highly numerate and health literate patients with diabetes were far more likely (a 34% difference in rates) to call their doctor when the hemoglobin A_1c_level was 8.4% than 7.1%.

### Perceived Usefulness of Test Results Displays

The 2 questions that measured perceived usefulness of the test results displays were highly correlated (*r*=.77), and the combined measure was highly reliable (Cronbach alpha=.87). Mean perceived usefulness was only 3.38 (SD 1.18) on a 1-5 scale, indicating a significant number of participants found these tables to be difficult to understand and/or not useful. Both numeracy and health literacy were positively correlated with ratings of perceived usefulness (numeracy: *r*=.32, *P*<.001; health literacy: *r*=.26, *P*<.001).

## Discussion

### Principal Results

A key reason why many patients want direct access to their medical test results is to verify which of their tests are okay and which are not. Unfortunately, our results suggest that many people find the task of identifying out-of-range values embedded in standard test result tables to be quite difficult. Perhaps more importantly, ability to accomplish this task appears highly related to both numeracy and health literacy skills. Participants with lower numeracy and health literacy skills were less than half as likely as those with higher numeracy/literacy abilities to identify hemoglobin A_1c_ levels as outside the reference range in a larger set of results, even though that test was specifically identified in the scenario as the reason for testing. Correctly identifying hemoglobin A_1c_ levels as out of range was, in turn, the single largest predictor of both perception of blood glucose control and intention to call one’s doctor in response to the elevated test results.

Health literacy and numeracy skills also appear to enable patients to know when they do or do not need to act in response to test results even after controlling for the effect of being able to correctly identify hemoglobin A_1c_ levels as out of range. Among study participants with diabetes (971/1812, 53.59%), increased health literacy was associated with lower intentions to call the doctor’s office for the (barely elevated) test result of hemoglobin A_1c_=7.1%. When the test result was the more substantially elevated hemoglobin A_1c_ value of 8.4%, it was the more numerate participants who were significantly more likely to call their doctor in response. In contrast, less numerate participants did not appear to recognize that the substantial jump in their hemoglobin A_1c_ results represented a trend worthy of immediate response.

This latter effect is particularly important, as it demonstrates the important distinction between patients knowing their test result numbers versus grasping the personal meaning of those data. For example, a patient with diabetes may use a patient portal to learn that her hemoglobin A_1c_ level changed from 10.1% to 9.3%, but have no idea that a change of less than 1 percentage point represents a significant reduction that corresponds to substantial health and risk reduction benefits. For this patient, knowing the numerical value of her test results did not ensure that she understood what those numbers implied or what actions she needed to consider. Her data were literally meaningless, and she is likely to ignore them in managing her health.

We deliberately studied reactions to laboratory test results among participants with and without diabetes, who would be expected to be more and less familiar with the types of data, respectively. Our results suggest that although familiarity with a metric such as hemoglobin A_1c_ levels is an important first hurdle for new patients, education about these measures is unlikely to be enough to achieve understanding for all patients. Those with low health literacy and low numeracy may require additional support, and interface design for laboratory results in patient portals should take these factors into account.

### Relationship to Prior Work

The generally welcome trend in recent years of people gaining access to their own test results has given rise to a common concern about the design of test results displays. In 2010, Wired Magazine ran a feature article titled “The Blood Test Gets a Makeover,” in which several designers were asked to develop “proof of concept” graphical test results reports [43]. These concept graphics used simple line graphs with clear, strong, color cues, reference points, and explanatory language to make multiple types of test results more meaningful to patients. In 2012, the US General Services Administration sponsored a test result design challenge that attracted over 230 entries [44]. These design initiatives demonstrate the widespread concern about this issue and show promise of improvement.

Unfortunately, the design concepts generated have yet to be studied rigorously to evaluate their effects on patient comprehension and activation. For example, do the high/low “flags” often included in clinician interfaces for EHRs (but conspicuously omitted in the patient format used by the major academic medical system we modeled our stimuli after) provide net benefit by clarifying out-of-range values, or do they cause net harm by increasing patient alarm about values that are not clinically concerning? Would use of a categorization system (perhaps with icons) that labeled results by potential harm, not just what is inside or outside the standard reference range values, be useful in guiding patient behavior? Would horizontal line displays or color coding help the less numerate or less literate patients be better able to derive meaning from their test results?

Recent research on risk communication suggests that well-designed visual displays can improve patient understanding of medical data, especially among those with lower numeracy skills [45-47]. Research also supports the supposition that including relevant reference standards beyond the “standard range” values in such displays is likely to make even unfamiliar test data more intuitively interpretable for patients [46,48,49]. Empirical research is needed to answer the preceding questions, thereby guiding the design of results displays to ensure that data are meaningful across levels of literacy and numeracy. In its absence, these barriers will continue to impede effective patient use of test result data (in electronic health records or elsewhere) to improve patient self-management and patient-provider communications.

Yet, it is worth asking ourselves: why are we giving patients these numbers? In many circumstances, patients’ informational goals would be addressed more directly by communications that highlight evaluative categories (eg, “poor,” “very high,” “borderline high”) over the specific numerical values. Both we and others have recently argued that precise numerical communications of health data can sometimes be counterproductive [50,51]. Our results suggest that patients with limited numeracy and health literacy skills may be particularly likely to benefit from alternate communication approaches that reinforce the critical “gist” messages [52] before presenting quantitative test result data.

### Limitations

Our findings are tempered by several important limitations. First, our study involved a hypothetical vignette and mock test results presented to people who knew they were taking a survey. The lack of personal relevance of these data may have inhibited participants’ motivation to seek out and identify the out-of-range values, and it is certainly possible that both perceptions of blood glucose control and intentions to call one’s doctor might be different if these were the patient’s own test results viewed in an actual EHR portal. Another limitation is that the study displayed all test results simultaneously on a single page (to facilitate their presentation within the survey engine and to allow us to test understanding, not recall), whereas many electronic health record systems only show 1 panel’s worth of results at a time. Although both of these limitations may affect the generalizability of our findings into actual clinical practice, this controlled experiment demonstrates the plausibility of literacy and numeracy concerns. Nonetheless, further research is clearly needed to study how well patients understand their own test results in a patient portal.

### Conclusions

Our results reinforce the critical role of health literacy and numeracy skills in enabling patients to take active roles in their health care. Being an “informed” patient requires more than having access to test results or being able to recite specific numbers. It means understanding what test data mean for evaluating one’s health status and how it should influence future health decisions or behaviors. Our data demonstrate that limited health literacy and numeracy are significant barriers to such knowledge translation tasks. Further research should investigate designs that help people better interpret the meaning of their numbers.

## Abbreviations

CBC: complete blood cell count
EHR: electronic health record
MCH: mean corpuscular hemoglobin
MCHC: mean corpuscular hemoglobin concentration
REALM: Rapid Estimate of Adult Literacy in Medicine
S-TOFHLA: Short Test of Functional Health Literacy in Adults
SNS: Subjective Numeracy Scale
SSI: Survey Sampling International
WBC: white blood cell
